# Perceptions of Family Health Strategy doctors and nurses on the promotion of adequate and healthy eating practices in light of the Dietary Guidelines, Santa Bárbara d’Oeste, 2022: qualitative study

**DOI:** 10.1590/S2237-96222025v34e20240456.en

**Published:** 2025-06-13

**Authors:** Hariane Thaine Bueno Rodrigues, Mariana Tarricone Garcia

**Affiliations:** 1 Secretaria Municipal de Saúde de Santa Bárbara d’Oeste, Setor de Nutrição, Santa Bárbara d’Oeste, SP, Brazil; 2 Secretaria de Estado da Saúde de São Paulo, Instituto de Saúde, Programa de Mestrado Profissional em Saúde Coletiva, São Paulo, SP, Brazil

**Keywords:** Dietary Guidelines, Food and Nutritional Health Promotion, Patient Care Team, Nutritional Policy, Qualitative Study, Guías alimentarias, Promoción de la Salud Alimentaria y Nutricional, Equipo de atención al paciente, Política nutricional, Investigación cualitativa

## Abstract

**Objective:**

To explore the perception of doctors and nurses from the Family Health Strategy in the municipality of Santa Bárbara d’Oeste/State of São Paulo regarding the promotion of adequate and healthy eating practices, in light of the Dietary Guidelines for the Brazilian Population.

**Methods:**

All doctors and nurses from the Family Health Teams were interviewed and the interviews were transcribed for content analysis. The thematic categories for analysis were defined a priori, having the Dietary Guidelines as a guide. The findings were discussed considering the Health Promotion framework.

**Results:**

Doctors (n=11) and nurses (n=9) were mostly female, between 36 and 45 years old and with 1 to 5 years of experience in the Family Health Strategy. Perceptions about their knowledge about food and health, autonomy in food choices, nutritional reductionism, convenience and cost appeared as challenges and were shown to be misaligned with the Dietary Guidelines. However, their perceptions about the consumption of ultra-processed foods and commensality were more in line with the Guidelines.

**Conclusion:**

Professionals perceive limitations in their knowledge about food and nutrition and the difficulties in carrying out actions to promote adequate and healthy eating, focusing on specific nutrients and restrictive prescriptions. The perception of the consumption of ultra-processed products as something harmful and the recognition of the influence of the context in which eating occurs on eating practices were observed. Despite the perception of obstacles to adequate and healthy eating as highlighted in the Dietary Guidelines, no strategies were suggested to overcome them.

## Resumen

**Objetivo:**

Explorar la percepción de médicos y enfermeros de la Estrategia de Salud de la Familia del municipio de Santa Bárbara d’Oeste/São Paulo sobre la promoción de prácticas alimentarias adecuadas y saludables, a la luz de la Guía Alimentaria para la Población Brasileña.

**Métodos:**

Se entrevistó a todos los médicos y enfermeros de los Equipos de Salud de la Familia y las entrevistas fueron transcritas para análisis de contenido. Los núcleos temáticos de análisis fueron definidos a priori, tomando como guía la Guía. Los hallazgos se discutieron a la luz del marco de promoción de la salud.

**Resultados:**

Los médicos (n=11) y enfermeros (n=9) eran en su mayoría mujeres, con edad entre 36 y 45 años y con 1 a 5 años de experiencia en la Estrategia de Salud de la Familia. Las percepciones sobre sus conocimientos acerca de la alimentación y la salud, la autonomía en la elección de alimentos, el reduccionismo nutricional, la conveniencia y el costo aparecieron como desafíos y se demostró que no estaban alineadas con la Guía. Sus percepciones sobre el consumo de alimentos ultraprocesados y la comensalidad estaban más en línea con la Guía.

**Conclusión:**

Los profesionales perciben limitaciones en sus conocimientos sobre alimentación y nutrición y dificultades para realizar acciones para promover una alimentación adecuada y saludable, presentando un enfoque en nutrientes específicos y prescripciones restrictivas. Se observó la percepción del consumo de productos ultraprocesados como algo nocivo y el reconocimiento de la influencia del contexto en el que se ingiere en las prácticas alimentarias. A pesar de la percepción de obstáculos para una alimentación adecuada y saludable resaltada en la Guía, no se sugirieron estrategias para superarlos.

**Ethical aspects**

This research respected ethical principles, having obtained the following approval data:

Research Ethics Committee: Instituto de Saúde de São Paulo

Opinion number: 5.636.208

Approval date: 12/9/2022

Certificate of Submission for Ethical Appraisal: 62525822.6.0000.5469

Informed Consent Form: Obtained from all participants prior to collection of samples.

## Introduction

Brazil is experiencing a significant increase in the prevalence of chronic non-communicable diseases, overweight and obesity, associated with changes in food consumption patterns, with a progressive and rapid increase in the consumption of ultra-processed foods (1). In parallel, the prevalence of food and nutritional insecurity in the Brazilian population is relevant (2). Poor diet is the leading cause of disability-adjusted life years (DALYs) lost, surpassing other risk factors such as smoking, overweight, physical inactivity, alcohol and drug use (3). Dealing with this scenario requires complex efforts from the SUS (Brazilian Unified Health System) and prioritization of public policies to address it (3). 

Established in 1999, the National Food and Nutrition Policy gave substance to food and nutrition actions in the SUS (4). By encouraging intersectoral actions, the purpose was to guarantee the quality of food consumed in the country, promote healthy eating practices, and prevent and control nutritional disorders (4). After a decade, the policy was revised and considered the epidemiological and nutritional transition processes and the changes in the food consumption profile and their repercussions (5). The updated version focused on nutritional care, integrated with other actions in the Health Care Networks, with Primary Health Care (PHC) organizing the actions, and assigning service professionals to carry out actions to promote adequate and healthy eating (6). The National Health Promotion Policy also presents the promotion of adequate and healthy nutrition as a priority, integrating health promotion, food and nutritional security and the guarantee of the human right to adequate food (7).

The Dietary Guidelines are one of the strategies for implementing an adequate and healthy eating, as they are official documents that define guidelines on healthy eating for the population (8). The first edition of the Dietary Guidelines for the Brazilian Population, launched in 2006, brought innovative discussions - such as the food system, food and nutritional security, food culture and commensality - and its content was based on the classification of foods according to their nutrient content, portions of food to be consumed and energy value of the diet (9). In 2014, the Guidelines introduced new concepts on the understanding of eating practices in the context of the food system, aligning with the current stage of the epidemiological and nutritional transition and based on the rights to health and adequate food (10). The document presents recommendations that do not address portion size and caloric value and classifies foods according to the extent and purpose of the processing to which they are subjected (10).

The Dietary Guidelines are a tool designed to support and encourage healthy eating practices aimed at the entire population, in addition to serving as a basis for policies, programs and initiatives aimed at promoting health and ensuring food and nutritional security for the population (10). Health professionals are fundamental in disseminating the Guidelines’ contents and must take ownership of actions to promote adequate and healthy eating in PHC (6). However, PHC professionals often do not have enough confidence in using the Guidelines to carry out these actions in their area of work (11-13). Thus, the implementation of the Guidelines in PHC presents challenges, even with recent efforts by the Ministry of Health, such as the publication of the Instruction Manual for the implementation of the Guidelines in PHC (14), the protocols for using the Guidelines (15) and the provision of the QualiGuia course at the SUS (Brazilian Unified Health System) Open University (16). 

Considering that the Family Health Strategy (ESF) was designed with the aim of reorganizing PHC to achieve the principles of universalization, equity and comprehensiveness in care and prioritize actions to promote, protect and recover health (17), it is essential to understand the perception of ESF professionals in relation to the promotion of adequate and healthy nutrition as a way of supporting actions to improve the quality of health services. Thus, the objective of this study was to explore the perception of doctors and nurses from the ESF in the municipality of Santa Bárbara d’Oeste - State of São Paulo on the promotion of adequate and healthy eating, in light of the principles, recommendations and obstacles referred to in the Guidelines. 

## Methods

### Study design, context and participants

Qualitative, exploratory and descriptive study conducted with doctors (n=11) and nurses (n=9) from the six basic health units with ESF teams in Santa Bárbara d’Oeste/State of São Paulo, with the purpose of investigating the experience of professionals in their daily work, seeking to understand issues of their own reality. The municipality has approximately 200,000 inhabitants, with PHC coverage of 41.7% and ESF coverage of 10.6%. Since the suspension of the Family Health and Primary Care Support Center (NASF-AB) in 2020, the PHC has not had a multidisciplinary team. Community health agents, nursing technicians/assistants, dental surgeons, specialist doctors, pharmacists, pharmacy technicians, social workers and oral health assistants also worked in the units.

The inclusion criterion for participating in the study was: working as a doctor or nurse in the municipality’s ESF teams. There was no exclusion criterion. These participants were selected because they were the professionals most involved in food and nutrition care in the units.

### Data collection

Data collection took place in November/December 2022 through interviews conducted by the author HTBR in person, which lasted approximately 30 minutes. The script was improved by a pilot study, conducted two months before data collection, which included interviews with a doctor and a nurse from an ESF team in a different municipality. Closed questions addressed professional and sociodemographic variables and open questions addressed the promotion of adequate and healthy eating in PHC (work routine, most frequent needs of users, approach, the role of agents in promoting adequate and healthy eating, implementation of actions to promote adequate and healthy eating). 

### Data analysis

Quantitative data were analyzed using descriptive statistics. The interviews were transcribed and coded to perform the Thematic Content Analysis, according to Bardin (18). The thematic categories for analysis were defined a priori, using the principles, recommendations and obstacles mentioned in the Guidelines as a guide. The thematic categories emerged from the interviewees’ statements. The analytical coding process used the semantic criterion to aggregate the responses and generate the recording units. 

After skimming the transcripts (pre-analysis), an exhaustive reading of the material and the analytical coding process began so that, based on the content of the interviewees’ statements, the identified themes could be assigned to the recording units (exploration of the material). Finally, the codes were grouped into thematic categories (treatment of results). The findings were discussed in light of the Health Promotion framework. The Guidelines are based on the concept of holistic health adopted by the Health Promotion, which considers social and collective factors, and not only the physical or biological condition of individuals (1,7), in addition to bringing principles, strategies and actions of Health Promotion, such as: healthy public policies, intersectorality, popular participation, creation of healthy environments, sustainability, development of skills, empowerment, personal autonomy, access to quality information, and reorientation of health services, among others (10).

## Results

The majority of participants (85.0%) were female and were between 36 and 45 years old (80.0%) and most had graduated 1 to 5 years ago (40.0%) and had worked in the ESF for 1 to 5 years (65.0%). There were more nurses (77.8%) who took subjects related to nutrition in their undergraduate studies than doctors (36.4%). Only two doctors have taken courses on food and nutrition lasting more than 20 hours in the last five years. All professionals reported dealing with issues related to food and nutrition in their routine at the ESF. (Table 1)

**Table 1:** Characterization of Family Health Strategy doctors and nurses participating in the study. Santa Bárbara d’Oeste, 2022 (n=20)

Variables	Doctors (n=11)	Nurses (n=9)	Total (n=20)
	n (%)	n (%)	n (%)
Gender			
Female	9 (81.8)	8 (88.9)	17 (85.0)
Male	2 (18.2)	1 (11.1)	3 (15.0)
**Age** (years)			
26–35	1 (9.1)	1 (11.1)	2 (10.0)
36–45	9 (81.8)	7 (77.8)	16 (80.0)
46–55	0 (0.0)	1 (11.1)	1 (5.0)
≥56	1 (9.1)	0 (0.0)	1 (5.0)
**Training time** (years)			
1–5	4 (36.4)	4 (44.4)	8 (40.0)
6–10	4 (36.4)	1 (11.1)	5 (25.0)
11-15	3 (27.3)	4 (44.4)	7 (35.0)
**Time working in Family Health Strategy** (years)			
1-5	7 (63.6)	6 (66.7)	13 (65.0)
6-10	4 (36.4)	1 (11.1)	5 (25.0)
11-15	0 (0.0)	2 (22.2)	2 (10.0)
**Professionals who took any nutrition course during their undergraduate degree**	4 (36.4)	7 (77.8)	11 (55.0)
**Professionals who took a course on food and nutrition (more than 20 hours) in the last 5 years**	2 (18.2)	0 (0.0)	2 (10.0)
**Professionals who took a course on food and nutrition (less than 20 hours) in the last 5 years**	2 (18.2)	2 (22.2)	4 (20.0)
**Professionals who address food and nutrition in their routine care**	11 (100.0)	9 (100.0)	20 (100.0)

Six thematic categories were identified, guided by the thematic categories of “principles”, “recommendations” and “obstacles”, addressed in the Guidelines, and one on “accountability for promoting adequate and healthy eating”, which emerged from the responses and proved to be relevant to the theme addressed in this study. 

Table 2 presents quotes that illustrate the thematic category “Guidelines Principles”. Participants expressed limitations in their knowledge about food and nutrition and difficulties in promoting adequate and healthy eating. They demonstrated a lack of confidence in providing guidance in certain areas and highlighted the need for support from specialists and further studies to deal with the topic in their routines. The need for more time to provide adequate guidance was also mentioned. One professional cited the lack of parameters regarding food portion sizes. The reduction of foods to their individual nutrients was present in the reports, with recommendations grouping foods according to their sources of macronutrients. Carbohydrate-rich foods have often been associated with the concept of “unhealthy”, without analyzing the amount of processing of these foods. Some reports attributed poor nutrition to users’ lack of knowledge about the nutritional content of foods. There was an association between healthy eating and dietary restrictions and the adoption of a prescriptive approach, believing that dietary guidelines should be followed by users. The lack of adherence to the eating behaviors they prescribed was attributed to a failure of the user to follow their guidelines as it was a matter of individual choice. 

**Table 2:** Excerpts from participants’ quotes that illustrate the thematic categories “Guidelines Principles”. Santa Bárbara d’Oeste, 2022 (n=20)

Thematic category: Principles of the Dietary Guidelinesa	
Thematic categories	Quotes
**Knowledge about food** **and health**	“*And then you need nutritional guidance, diet, and we have a limitation in terms of what we can explain, what we can advise, and so we need the support of specialists. (…) And it takes a lot of time for you to provide guidance, you also have to study a little more about how to educate the patients, what they can eat, what they can’t. (...) And we don’t always know the nutritional part of vitamins, nutrients, that the person also needs to have a balanced diet. (…) The cholesterol part, triglycerides, the main foods. There are foods that we know are a little fattier, and there are others that could be harmful, and we don’t even imagine. So having this support is very important*.” (MD11).
	“(...) *Children too, pregnant women too... what shocks me most is this issue with diabetics... I think that in childcare as well, in introducing food, I think we lose a little, sometimes even due to lack of knowledge, in presenting it in a good way, even due to time issues. I think it’s also due to the lack of training, a topic that I don’t master, I don’t discuss it much, right, I don’t talk much about it, I think that’s why*” (MD08).
	“*I have, like, a specific topic, I have a lot of difficulty with diabetics. I really have trouble with them. (...) Ah, obesity is an easier topic for us to address, right? Maybe dyslipidemias too, right? But I think we have difficulty with chronic diseases related to poor diet*” (MD03).
	“*I still have difficulty with child nutrition* (...) *Children, yes, this is not easy for me. (...) Pregnant women too, I think I’d like to work better with this group, in my training, tin college we even study this, but it’s not, it’s there, right? Just superficially*” (ENF02).
	“*But I have my difficulties, right? The case of portions*, right?” (ENF02).
	“*Of course I don’t have that much knowledge, so I say what I... As far as I can go*, right?” (ENF06).
**Nutritional reductionism**	“*Now skip to lunch, and then that dish, right, that has a lot of carbohydrates, then you start finding dietary mistakes*” (ENF02).
	“*It’s hard to find just one patient that eats properly. It is very difficult* (...) *But then you’ll look for what he eats: ‘What did you eat this morning?’. ‘Ah, my bread’. ‘But the French bread?’. ‘Yeah, French bread*’” (ENF09).
	“*Here, they eat badly, they don’t know what to eat, they don’t know what carbohydrates are, what proteins are, what… They don’t know. They don’t know. (...) Like, they don’t know that bread increases diabetes*.” (MD05).
	“*I never talk about carbohydrates because sometimes they don’t know, but when I tell them that they can’t eat rice and potatoes, rice and cassava together, they: “Oh, really?”’ (...) Another thing they are surprised about is when I say that they can’t eat too much flour, rice, beans and flour. But what am I going to eat? (...) So, and sometimes, it’s a basic thing like that, like I said, right? So flour, cassava flour, right? And they eat everything, but because they don’t know it’s carbohydrates*” (MD07).
	“*Patient with dyslipidemia… I provide guidance on regular physical activity, walking, dietary control… reducing the intake of sugars, fats, starchy foods, increasing water intake, increasing the intake of fiber, vegetables, legumes. (...) Basically, to reduce fats and starchy foods*” (MD04).
	“*We speak in a very general way: avoid pasta, avoid sweets, avoid foods with a high fat content*” (MD06).
**Autonomy in food choices**	“*Food restriction is not easy for anyone, you need to change your habits*, right?” (MD10).
	“*Generally, at every appointment I explain what he can and cannot eat*” (MD07).
	“*Food reeducation is only there to help, right? When the patient understands and accepts what we are saying*” (ENF08).
	“*So it’s very difficult, they don’t follow, you educate them… But they have a lot of difficulty* [in following the instructions from healthcare professionals]” (ENF02).
	“*I realize that when we talk to them about food, they listen. (...) There are some who accept it. There are some who don’t accept it. (...) But it is really difficult to get them to really adhere to it*.” (ENF09).
	“*What is the first thing that they* [users] *say, right? ‘Wow, now I have to stop eating everything*, right?’” (ENF07).
	“*They’d rather stuff themselves with insulin than take care of their diet at home, you know? (...) Unfortunately, the allopathic conception is very cultural, so in their heads I think they think like this: Ah, but uh, for example, I’m not going to be here just craving for this, I’m going to eat this and then I’ll increase the insulin by 5 to 10 units, I don’t know*” (ENF03).

^a^Dietary Guidelines Principles: Food is more than just nutrient intake; Recommendations about proper food must be in tune with the times; Adequate and healthy food comes from a socially and environmentally sustainable food system; Different types of knowledge generate the knowledge for formulating Dietary Guidelines; Dietary Guidelines increase autonomy in food choices (10).

Quotes that illustrate the categories defined according to the “Guideline Recommendations” are presented in Table 3. The professionals reported that they provided guidance on the importance of reducing the consumption of “processed” foods, as they recognized that consumption was high and harmful to health. Although the term “ultra-processed” was rarely mentioned, interviewees seemed to recognize these foods. It is worth mentioning that some professionals stated that in their practices they encouraged greater consumption of fruits, rice and beans and the replacement of processed meats with eggs. The interviewees highlighted the pleasure involved in eating and the importance of eating mindfully, and of having shared meals, and an appropriate environment, as well as the social context of eating, for both adults and children. The influence of the context in which eating occurs and family eating practices were recognized as factors in promoting healthy eating.

**Table 3:** Excerpts from participants’ quotes that illustrate the thematic categories “Dietary Guidelines Recommendations”, Santa Bárbara d’Oeste, 2022 (n=20)

Thematic category Dietary Guidelines Recommendations^a^	
Thematic categories	Quotes
**Consumption of ultra-processed foods**	“*What I advise most, what I talk and focus a lot on, is to avoid processed foods, because here they eat a lot of mortadella, sausages, they really like it. (...) I advise more in general terms… to avoid processed foods, even crackers that they think are healthy, eat and focus more on fruits, rice and beans*” (ENF02).
	“*They eat a lot of processed foods, they really do*…” (MD03).
	“*So it turns out that they consume more processed foods, a lot of ready-made things, and at least that’s what I observe when I ask*” (MD09).
	“*I always insist that they must try not to eat too many sausages. (...) Okay, you can’t buy meat? Replace with egg. Because sausage is full of salt, sodium, flavor enhancers*” (ENF04).
Commensality	“*So, we know that eating is a moment of pleasure. You have to pay attention to what you are eating, feel the pleasure of eating. There, watching television, you become completely distracted. You end up eating more sometimes and not paying attention. This robs you of the pleasure that we know exists in eating*” (ENF02).
	“*If the adult eats something, the child will eat it too, right? At school itself, you see. The child has a different diet in school. Why do you eat? He sees everyone eating, he simply eats*” (ENF07).
	“*Eating with family, right? (...) Family meals are important, right? So that’s essential for me*.” (MD10).

^a^Dietary Guidelines Recommendations: Make food in natura or minimally processed foods the basis of your diet; Use oils, fats, salt and sugar in small quantities when seasoning and cooking food and creating culinary preparations; Limit the use of processed foods, consuming them in small quantities, as ingredients in culinary preparations or as part of meals based on processed foods in natura or minimally processed; Avoid ultra-processed foods; Always prefer foods in natura or minimally processed and culinary preparations to ultra-processed foods; Eat regularly and mindfully; Eat in appropriate environments; Eat with company (10).

The “Obstacles to adherence to the Guidelines’ recommendations” guided the definition of the categories whose citations are presented in Table 4. The shorter time and simpler preparation or lack of preparation of ultra-processed foods were understood as greater convenience for consumers, even though reports associated the preparation of home-cooked meals with healthier and tastier foods. The narratives about the practicality offered by ultra-processed foods stood out, which, for the interviewees, outweighed the effort required to prepare healthy meals, especially given the overload of other household chores and self-care or care for others. Lack of time was reported as a significant factor impacting food choices, which was one of the main reasons that led users to prioritize quick and practical meals, often opting for ultra-processed and fast food. The high cost of food was identified as a major barrier to users adopting a healthy diet. Respondents reported people’s propensity to choose cheaper and more easily accessible, albeit less nutritious, foods, such as ultra-processed foods. Professionals recognized users’ financial difficulties as a significant challenge to promoting adequate and healthy eating.

**Table 4:** Excerpts from participants’ quotes that illustrate the thematic categories “Obstacles to adherence to the Dietary Dietary Guidelines’ Recommendations”. Santa Bárbara d’Oeste, 2022 (n=20)

Thematic category: Obstacles to adherence to the Dietary Guidelines Recommendationsa	
Thematic categories	Quotes
Convenience	“*Imagine having to do it every day, take care of the house, and still take care of the grandchildren, and still do this* [prepare meals], *it’s really not that easy*, right? (ENF07).
	“*I think that eating healthier is a little more work, right? So you have to wash a salad, you have to make rice, cook beans, and so on. Not everyone wants to do it, right? Even though it’s the tastiest thing, but that’s a habit, right? It’s a habit. If that family doesn’t have that habit, they’ll do whatever they can, right? Sometimes, at the supermarket, you look at the person’s cart, at that pile of noodles, and you see what they’re going to eat. It’s easy*, right?” (ENF08).
	“*We see some cases of obese children who use a lot of industrialized products.(...) Because it’s an easier thing for parents, right? They don’t have to go to the kitchen to prepare it, so they go to the grocery store and buy it*” (ENF06).
	“*Of course, many people will rather go for food which is quick, fast food, stuffed cookies*” (ENF02).
	“*So it’s easier, I go to the bakery, it’s ready, I grab it and it’s done*” (ENF03).
	“*They prioritize easy, quick meals... ‘But what did you eat today?’ ‘So, ah, I just ate some noodles*’” (ENF08).
	“*But you see that today, people are time-pressed, mothers have little time because they work, they have to work twenty-nine shifts, right, and then it ends up being easier, it ends up being ultra-processed foods, like that, in the vast majority of cases*” (MD08).
Cost	“*I think they end up consuming more processed foods because of the price, practicality and so on.(...) We recommend everything that is healthier, but sometimes the patient does not have the financial means to pay for a healthier diet*.” (MD09).
	“*So sometimes we even provide guidance on the issue of balanced eating, food groups, but often what we hear is that we don’t have the financial means to have salad, fruit, vegetables, and they end up having diets very carbohydrate-based and rarely protein*” (MD06).
	“*There are people who have difficulty eating vegetables, they say it is expensive, I don’t think it is, but they say it is, so it is the industrialized product* [which replaces it]” (MD03).
	“*Processed food, as you know, has a much lower cost and many of them prioritize these foods. So, it’s more a question of practicality, ease of access, which they themselves say often makes taking care of their own diet unfeasible. So, it’s that question, right, of the double-edged sword. It is easy to acquire, but it is also of low quality. So, I realize that this is one of the most aggravating factors in relation to criticism of the quality of the food*.” (MD02).
	“*So, it is even difficult to talk about good nutrition for a patient whose income is what he receives from Bolsa Família* (*a federal monetary aid to poor families). So, this is a major obstacle for my population*” (MD01).

^a^Obstacles to adherence to the Dietary Guidelines’ recommendations: Information; Offer; Cost; Cooking skills; Time; Advertising (10).

Table 5 shows quotes that reflect a diverse view on the shared responsibility for promoting adequate and healthy nutrition. There was consensus on the need to involve the team, but they also pointed out challenges such as lack of time and knowledge, insecurity and the need for training on this topic. There was a view that the nutritionist was the appropriate professional to carry out this task, therefore, the other professionals promoted adequate and healthy eating as best they could, given their limitations. The interviewees revealed that they prioritized medical-curative guidelines in light of user demands, and the approach to food and nutrition was not a common practice in PHC. One report highlighted the potential of home visits as a strategy for promoting adequate and healthy eating.

**Table 5:** Excerpts from quotes from participants that illustrate the thematic category “Accountability for promoting adequate and healthy nutrition”. Santa Bárbara d’Oeste, 2022 (n=20)

Thematic category: Accountability for promoting adequate and healthy nutrition	
Thematic category	Quotes
**Accountability for promoting adequate and healthy eating**	“*It doesn’t have to be just the nutritionist, because the nutritionist is one, right? One or two. And she’s not in the community every day. She can’t, right? She can’t be at so many places at once. So, she could you pass on this responsibility, this... It’s not responsibility, this commitment, but with proper training*” (ENF06).
	“*We know that the SUS* (*Brazilian Unified Health System) has a resource management, so there’s no point in us dreaming just for the sake of it, thinking that we’ll have a nutritionist at each UBS, right? And even if we had one, she wouldn’t be able to provide guidance to all of her patients, a nutritionist. So, in the end the doctor, the nurse, they will have to have this guidance too*” (MD06).
	“*All of us as a team have to have this focus, right, this focus on nutrition as well. Yes, this is not always done, right, because unfortunately we end up directing it towards that momentary pathology that the patient comes with a complaint about, right, and the nutritional issue is not always addressed by this group of professionals that treats the patient, right? So, if we could really highlight the importance of this, not only of the doctor, but of other professionals, in this sense, I think it would be a gain, right, for the population... (...) the more professionals participate in this, in this promotion, the more gain we will have, right, in the future for the population as well*” (MD02).
	“*Look, what I can do more incisively in relation to patients, I can do, within my limitations as a doctor, after all I am not a nutritionist*” (MD01).
	“*I think we can address it a little, I always try to address it, but it’s like, we do it in a very superficial way, I think. And even, perhaps, due to lack of training as well, anyway, I think that, yes, I do, but I think it could improve and the demand could increase for what I offer*.” (MD08).
	“*So, the nurse’s action is, unfortunately, more curative, right? Whe the tests are already altered, when there is already some disability, for example, there is already some injury to the foot, there is already something... Unfortunately, I don’t have... I don’t see much resolution, results. (...) And it’s so simple, it’s such an obvious thing, but I can’t convey it to the patient* [dietary guidance]. (...) *We understand that, that’s frustrating, that’s what frustrates us, you know? It’s frustrating. This is frustrating. And you know your responsibility, right? It goes without saying, but it’s something that’s really frustrating*.” (ENF07).
	“*But it’s an obstacle, you know? Talking about nutrition in primary care is complicated. (...) It’s not that simple because you see that it’s not a habit. I told you this so you can see that it is not a habit for nurses to talk about it*.” (ENF04).
	“*It all starts at the patient’s home, as the strategy dictates, we make a visit, we evaluate, we talk and I tell the patient, sometimes I will make a visit, I will go there to see what is in the fridge, if he authorizes me to do this, of course, right? Look, let’s try to have a more appropriate diet? So I have this access*.” (ENF02).
	“*I don’t have time to work on this diet, right? It’s much easier for me to prescribe the medication*.” (MD03).

## Discussion

To our knowledge, this is the first qualitative study with ESF professionals on the promotion of adequate and healthy eating in light of the current Dietary Guidelines. The study stands out for capturing the perspectives of participants and the knowledge acquired through their experiences with the promotion of adequate and healthy eating, allowing the exploration of information that emerged from the participants’ reflections on their experiences, encompassing their knowledge and practices. Even after a decade, the concepts of the second edition of the Guidelines have not been fully incorporated by the municipality’s ESF teams. It is worth noting that the period preceding this research was marked by the COVID-19 pandemic, when activities related to the pandemic were prioritized and training in other areas of the municipality, such as food and nutrition, was hampered (19). 

As evidenced in this study, professionals may feel unprepared to provide advice on food and nutrition (20) and, thus, provide superficial dietary guidance (21-22). This knowledge gap contributes to difficulties and insecurities in providing dietary guidance (21). The offer of training processes to promote adequate and healthy eating, aimed at both workers and managers, is essential to enable true empowerment and promote greater autonomy in decision-making (7). These processes should address tools for individuals and communities to develop self-care capabilities and act on environmental factors that influence their health (10). The locus of the study – the ESF – proposes a care model that considers the physical and social context, with the expansion of professionals’ understanding of the living and health conditions of communities, the health-disease process and the importance of interventions that transcend curative practices (17). 

In this study, professionals referred to the food groups according to their nutritional composition, emphasizing specific nutrients, in disagreement with the Guidelines. This perspective has been widely disseminated and used in actions to promote adequate and healthy eating (23) since the publication of the first edition of the Guidelines (9) and has become very popular, with recommendations on caloric intake and food portions, concepts brought by the participants of this study. The emphasis on isolated nutrients was observed, especially in relation to carbohydrates which, regardless of the level of processing, were seen as foods to be avoided. One of the fundamental principles of the current Guidelines (“Food is more than nutrient intake”) emphasizes a comprehensive approach to the concepts of food and health, going beyond the physiological and biological dimensions (10) and aligning with the Health Promotion framework. The Guidelines incorporate social, economic, political and cultural components and emphasize the promotion of autonomy in food choices and practices, engagement in actions that strengthen self-care and dialogue between individuals, health professionals and managers, valuing respect for desires, differences and subjectivities (1). Furthermore, participants associated healthy eating with restrictive diets and the adoption of prescriptive approaches, contrary to what the Guidelines recommend. By understanding food in a comprehensive way and its connection with health and well-being, the Guidelines consider, in addition to nutrients and foods, their combinations, forms of preparation and the cultural and social aspects of food (10).

The consumption of ultra-processed (or “industrialized”) foods was noted with concern by professionals. The growing advancement of food science techniques on a global scale has resulted in greater production and consumption of ultra-processed foods, including in Brazil (1,24-26), and the presence of these foods in users’ routines was noticed by those interviewed. Excessive consumption of these products is one of the main factors contributing to the global epidemic of obesity, diabetes and other chronic non-communicable diseases (1,26-27). 

The convenience of ultra-processed and fast foods was perceived as a strategy to save time and reduce the effort required to cook. In fact, these products are intentionally designed to be practical, requiring little or no preparation, with a long shelf life and, often, not requiring utensils, which makes them easy to consume in any context (28-29). Participants in our study perceived the lack of time for planning and preparing meals and easy access to ultra-processed foods as challenges, in line with the Guidelines, which recognizes that the devaluation of food preparation, the overload of daily tasks and the greater availability of ultra-processed foods are important obstacles to adopting an adequate and healthy diet (10). Preparing meals at home, although time-consuming, is essential for healthy eating practices, and this must be accompanied by an equitable division of household tasks to avoid overloading women (30). It is essential that healthcare professionals integrate these discussions into their PHC practices (10).

Our study highlighted the low cost and easy access to ultra-processed foods and the financial inaccessibility of “healthy” foods by users as significant challenges in the current food context. The food industry designs and markets these products with the aim of maximizing profitability, which is reflected in their economic accessibility (29). This reduced cost encourages the consumption of ultra-processed foods to the detriment of healthier options (28). In Brazil, a diet based on foods in natura or minimally processed foods is still more economical than a diet based on ultra-processed foods (31). However, it is estimated that ultra-processed foods could become even more accessible than healthy options by 2026 (32). No strategy was used by the participants to instruct users on how to save money on natural and minimally processed foods. Understanding the role of the food environment, considering both physical and financial access to food, is essential to address the barriers that individuals face in adhering to the Guidelines’ recommendations (1).

The act of eating and its dimensions were highlighted in this study. Commensality plays a crucial role in promoting healthy eating habits (10) and it has been recognized by professionals as an important factor, with an emphasis on shared meals and family eating practices, which have a relevant effect on children’s food choices, shaping their eating patterns (28). This perspective, highlighted by the interviewees, is in line with what in prescribed by the Guidelines and supported by multiple scientific evidence, which highlights, for example, the negative impact on food consumption of environmental distractions and activities carried out simultaneously with the act of eating, such as watching television or using computers or cell phones during meals, reducing the perception of satiety signals (1). From the perspective of Health Promotion, commensality involves coexistence, well-being, pleasure and happiness, which are considered in the construction and experience of health of individuals in their daily lives (7,10,33). 

It is important to highlight the participants’ self-perception as agents responsible for promoting adequate and healthy eating and the recognition of the importance of interprofessional collaboration, as well as their understanding that this action should not be attributed exclusively to a single professional. The implementation of the guideline for promoting adequate and healthy eating is the responsibility of SUS (Brazilian Unified Health System) managers and professionals, in partnership with other actors and sectors (10) and the responsibility for food and nutrition care must be shared among the PHC team (6). However, the lack of knowledge, security and training, highlighted in this study, are challenges that compromise the effectiveness of promoting adequate and healthy eating in PHC. PHC services are often marked by fragmentation of care and insufficient clinical accountability as a result of the organization of work and decision-making processes (17). This may result in the adoption of pharmaceutical approaches, seen as faster and more convenient (34), as found in this study. Comprehensiveness is a pillar in the construction of the SUS (Brazilian Unified Health System), and one of its dimensions refers to the priority of promotion and preventive actions, reinforcing a critical stance and the rejection of a health model focused on specialization and medicalization (17).

As probable limitations, we can mention the fact that the interviews were conducted by the person technically responsible for the nutrition service in the municipality, which may generate a bias, intentional or not, in the participants’ responses. To reduce this bias, the interviewer guaranteed confidentiality and anonymity, developed a good relationship with the participants and promoted a respectful and non-judgmental atmosphere. The choice to restrict study participants to doctors and nurses is justified by their responsibility for excessive referrals to nutritionists in specialized care and their relevance in nutritional counseling in the municipality’s PHC.

We conclude that perceptions regarding knowledge about food and health, autonomy in food choices, nutritional reductionism, convenience and cost diverge from the recommendations established in the Guidelines. Perceptions about the consumption of ultra-processed foods and commensality are in line with the principles and recommendations of the Guidelines. Since the PHC is an environment conducive to promoting adequate and healthy eating, health professionals need to be adequately prepared for this role. In addition to the need to qualify these workers, the implementation of multidisciplinary teams can be a strategy to expand the offer of actions to promote adequate and healthy eating, in addition to contributing to the effective implementation of the Guidelines in the ESF.

## Data Availability

Data related to this study are available at https://doi.org/10.7910/DVN/MZE9PM.
