# Supplementary material for: Perceptions of Family Health Strategy doctors and nurses on the promotion of adequate and healthy eating practices in light of the Dietary Guidelines, Santa Bárbara d’Oeste, 2022: qualitative study
Source: Epidemiol Serv Saude. 2025 Jun 13;34:e20240456. doi: 10.1590/S2237-96222025v34e20240456.en (PMC12176445; doi:10.1590/S2237-96222025v34e20240456.en)
Supplement: Translation

# Translation: Percepções de médicos e enfermeiros da Estratégia Saúde da Família sobre a promoção de práticas alimentares adequadas e saudáveis à luz do Guia Alimentar, Santa Bárbara d’Oeste, 2022: estudo qualitativo

**DOI:** 10.1590/S2237-96222025v34e20240456.pt

**Authors:** Hariane Thaine Bueno Rodrigues, Mariana Tarricone Garcia

**Affiliations:** 1 Secretaria Municipal de Saúde de Santa Bárbara d’Oeste, Setor de Nutrição, Santa Bárbara d’Oeste, SP, Brazil; 2 Secretaria de Estado da Saúde de São Paulo, Instituto de Saúde, Programa de Mestrado Profissional em Saúde Coletiva, São Paulo, SP, Brazil

**Keywords:** Guias Alimentares, Promoção da Saúde Alimentar e Nutricional, Equipe de Assistência ao Paciente, Política Nutricional, Pesquisa Qualitativa

## Abstract

**Objetivo:**

Explorar a percepção de médicos e enfermeiros da Estratégia Saúde da Família do município de Santa Bárbara d’Oeste/São Paulo sobre a promoção de práticas alimentares adequadas e saudáveis, à luz do *Guia Alimentar para a População Brasileira*.

**Métodos:**

Foram entrevistados todos os médicos e enfermeiros das Equipes de Saúde da Família e as entrevistas foram transcritas para a realização da análise de conteúdo. Os núcleos temáticos de análise foram definidos a priori, tendo como orientador o Guia. Os achados foram discutidos à luz do referencial da Promoção da Saúde.

**Resultados:**

Os médicos (n=11) e enfermeiros (n=9) eram, em sua maioria, do sexo feminino, entre 36 e 45 anos e de 1 a 5 anos de atuação na Estratégia Saúde da Família. As percepções sobre seus conhecimentos sobre alimentação e saúde, autonomia nas escolhas alimentares, reducionismo nutricional, conveniência e custo apareceram como desafios e se mostraram desalinhadas com o Guia. Já suas percepções sobre o consumo de ultraprocessados e comensalidade apresentaram mais alinhamento ao Guia.

**Conclusão:**

Os profissionais percebem limitações em seu conhecimento sobre alimentação e nutrição e dificuldades em realizar ações de promoção da alimentação adequada e saudável, apresentando um enfoque em nutrientes específicos e prescrições restritivas. Foi observada a percepção do consumo de produtos ultraprocessados como algo prejudicial e o reconhecimento da influência do contexto em que ocorre a alimentação nas práticas alimentares. Apesar da percepção dos obstáculos à alimentação adequada e saudável como apontados no Guia, não foram apontadas estratégias para superá-los.

**Aspectos éticos**

A presente pesquisa respeitou os princípios éticos, obtendo os seguintes dados de aprovação:

Comitê de ética em pesquisa: Instituto de Saúde de São Paulo

Número do parecer: 5.636.208

Data de aprovação: 12/9/2022

Certificado de apresentação de apreciação ética: 62525822.6.0000.5469

Registro do consentimento livre e esclarecido: Obtido de todos os participantes antes da coleta.

## Introdução

O Brasil vivencia o aumento expressivo da prevalência de doenças crônicas não transmissíveis, do excesso de peso e da obesidade, associados às mudanças nos padrões de consumo alimentar, com um progressivo e rápido aumento no consumo de produtos ultraprocessados (1). Em paralelo, é relevante a prevalência de insegurança alimentar e nutricional na população brasileira (2). A dieta inadequada é a principal causa de anos de vida perdidos ajustados por incapacidade (DALYs), superando outros fatores de risco, como tabagismo, excesso de peso, inatividade física, uso de álcool e drogas (3). Lidar com este cenário exige esforços complexos do Sistema Único de Saúde (SUS) e priorização de políticas públicas para seu enfrentamento (3). 

Estabelecida em 1999, a Política Nacional de Alimentação e Nutrição deu materialidade às ações de alimentação e nutrição no SUS (4). Com estímulo a ações intersetoriais, teve o propósito de garantir a qualidade dos alimentos consumidos no país, promover práticas alimentares saudáveis, prevenir e controlar distúrbios nutricionais (4). Após uma década, a política foi revisada e considerou os processos de transição epidemiológica e nutricional e as mudanças no perfil de consumo alimentar e suas repercussões (5). A versão atualizada teve foco na atenção nutricional integrada às demais ações nas Redes de Atenção à Saúde, tendo a Atenção Primária à Saúde (APS) como ordenadora das ações, e atribuindo aos profissionais dos serviços a realização de ações de promoção da alimentação adequada e saudável (6). A Política Nacional de Promoção da Saúde também apresenta a promoção da alimentação adequada e saudável como um tema prioritário, integrando a promoção da saúde, a segurança alimentar e nutricional e a garantia do direito humano à alimentação adequada (7).

Os guias alimentares são uma das estratégias para implementação da diretriz de promoção da alimentação adequada e saudável, se tratando de documentos oficiais que definem diretrizes sobre alimentação saudável para a população (8). O primeiro *Guia Alimentar para a População Brasileira*, lançado em 2006, trouxe discussões inovadoras - como o sistema alimentar, segurança alimentar e nutricional, cultura alimentar e comensalidade - e seu conteúdo era baseado na classificação dos alimentos segundo seus teores de nutrientes, porções de alimentos a serem consumidos e valor energético da dieta (9). Em 2014, o Guia trouxe novos conceitos sobre o entendimento das práticas alimentares dentro do contexto do sistema alimentar, alinhando-se ao atual estágio da transição epidemiológica e nutricional e tendo por pressupostos os direitos à saúde e à alimentação adequada (10). O documento apresenta recomendações que não abordam tamanho de porções e valor calórico, e classifica os alimentos segundo a extensão e a finalidade do processamento a que são submetidos (10).

O Guia é uma ferramenta destinada a apoiar e incentivar práticas alimentares saudáveis direcionado a toda a população, além de servir de base para políticas, programas e iniciativas que voltados à promoção da saúde e à garantia da segurança alimentar e nutricional da população (10). Os profissionais são fundamentais na disseminação do conteúdo do Guia e devem se apropriar das ações de promoção da alimentação adequada e saudável na APS (6). Contudo, é comum que os profissionais da APS tenham pouca segurança na utilização do Guia para realizar essas ações em seu território de atuação (11-13). Assim, a implementação do Guia na APS apresenta desafios, mesmo com os esforços recentes do Ministério da Saúde, como a publicação do Manual Instrutivo para a implementação do Guia na APS (14), dos protocolos de uso do Guia (15) e a disponibilização do curso QualiGuia na Universidade Aberta do SUS (16). 

Considerando que a Estratégia Saúde da Família (ESF) foi concebida no intuito de reorganizar a APS para alcançar os princípios de universalização, equidade e integralidade no cuidado e priorizar ações de promoção, proteção e recuperação de saúde (17), é primordial compreender a percepção de profissionais da ESF em relação à promoção da alimentação adequada e saudável como forma de subsidiar ações para melhorar a qualidade dos serviços de saúde. Assim, o objetivo deste estudo foi explorar a percepção de médicos e enfermeiros da ESF do município de Santa Bárbara d’Oeste - São Paulo sobre a promoção da alimentação adequada e saudável, à luz dos princípios, recomendações e obstáculos referidos no Guia. 

## Métodos

### Desenho do estudo, contexto e participantes

Estudo qualitativo, do tipo exploratório e descritivo conduzido com médicos (n=11) e enfermeiros (n=9) das seis unidades básicas de saúde com equipes de ESF de Santa Bárbara d’Oeste/São Paulo, com a finalidade de investigar a experiência dos profissionais em seu cotidiano de trabalho, buscando compreender questões da sua própria realidade. O município possui cerca de 200 mil habitantes, com cobertura de APS de 41,7% e de ESF de 10,6%. Desde a suspensão do Núcleo de Apoio à Saúde da Família e Atenção Básica (NASF-AB) em 2020, a APS não conta com equipe multiprofissional. Nas unidades atuavam também agentes comunitários de saúde, técnicos/auxiliares em enfermagem, cirurgiões dentistas, médicos especialistas, farmacêuticos, técnicos em farmácia, assistentes sociais e auxiliares em saúde bucal.

Os critérios de inclusão para participar do estudo foram: atuar como médico ou enfermeiro em equipes de ESF do município. Não houve critério de exclusão. Estes participantes foram selecionados por serem os profissionais mais envolvidos com os cuidados em alimentação e nutrição nas unidades.

### Coleta de dados

A coleta de dados ocorreu em novembro/dezembro de 2022 por meio da condução de entrevistas realizadas pela autora HTBR presencialmente, que duraram cerca de 30 minutos. O roteiro foi aprimorado por estudo-piloto, conduzido dois meses antes da coleta de dados, que contemplou a entrevista de um médico e um enfermeiro de uma equipe de ESF de um município distinto. Questões fechadas abordaram variáveis profissionais e sociodemográficas e questões abertas abordaram a promoção da alimentação adequada e saudável na APS (rotina de trabalho, necessidades mais frequentes dos usuários, forma de abordagem, o papel de agentes de promoção da alimentação adequada e saudável, realização das ações de promoção da alimentação adequada e saudável). 

### Análise dos dados

Os dados quantitativos foram analisados utilizando a estatística descritiva. As entrevistas foram transcritas e codificadas para a realização da Análise de Conteúdo Temática, segundo Bardin (18). Os núcleos temáticos de análise foram definidos a priori, tendo como orientadores os princípios, recomendações e obstáculos referidos no Guia. As categorias temáticas emergiram das falas dos entrevistados. O processo analítico de codificação utilizou o critério semântico para agregar os discursos e gerar as unidades de registro. 

Após a leitura flutuante das transcrições (pré-análise), iniciou-se a leitura exaustiva do material e o processo analítico de codificação para, a partir do conteúdo das falas dos entrevistados, serem atribuídos temas identificados para as unidades de registro (exploração do material). Por fim, foi feito o agrupamento dos códigos em categorias temáticas (tratamento dos resultados). Os achados foram discutidos à luz do referencial da Promoção da Saúde. O Guia está baseado no conceito de saúde integral adotado pela Promoção da Saúde, que considera os fatores sociais e coletivos, e não somente a condição física ou biológica dos indivíduos (1,7), além de trazer princípios, estratégias e ações da Promoção da Saúde, como: políticas públicas saudáveis, intersetorialidade, participação popular, criação de ambientes saudáveis, sustentabilidade, desenvolvimento de habilidades, empoderamento, autonomia pessoal, acesso a informações de qualidade, reorientação dos serviços de saúde, entre outros (10).

## Resultados

A maioria dos participantes (85,0%) era do sexo feminino e tinha entre 36 e 45 anos (80,0%) e a maior parte tinha de 1 a 5 anos de formados (40,0%) e de 1 a 5 anos de atuação na ESF (65,0%). Houve mais enfermeiros (77,8%) que cursaram disciplinas relacionadas à nutrição na graduação do que médicos (36,4%). Apenas dois médicos realizaram cursos sobre alimentação e nutrição com mais de 20 horas nos últimos cinco anos. Todos os profissionais relataram tratar de questões relacionadas à alimentação e nutrição em sua rotina na ESF. (Tabela 1)

**Tabela 1:** Caracterização dos médicos e enfermeiros da Estratégia Saúde da Família participantes do estudo. Santa Bárbara d’Oeste, 2022 (n=20)

Variáveis	Médicos (n=11)	Enfermeiros (n=9)	Total (n=20)
	n (%)	n (%)	n (%)
Sexo			
Feminino	9 (81,8)	8 (88,9)	17 (85,0)
Masculino	2 (18,2)	1 (11,1)	3 (15,0)
**Idade** (anos)			
26–35	1 (9,1)	1 (11,1)	2 (10,0)
36–45	9 (81,8)	7 (77,8)	16 (80,0)
46–55	0 (0,0)	1 (11,1)	1 (5,0)
≥56	1 (9,1)	0 (0,0)	1 (5,0)
**Tempo de formação** (anos)			
1–5	4 (36,4)	4 (44,4)	8 (40,0)
6–10	4 (36,4)	1 (11,1)	5 (25,0)
11-15	3 (27,3)	4 (44,4)	7 (35,0)
**Tempo de atuação na Estratégia Saúde da Família** (anos)			
1-5	7 (63,6)	6 (66,7	13 (65,0)
6-10	4 (36,4)	1 (11,1)	5 (25,0)
11-15	0 (0,0)	2 (22,2)	2 (10,0)
**Cursaram alguma disciplina de nutrição durante a graduação**	4 (36,4)	7 (77,8)	11 (55,0)
**Participaram de algum curso sobre alimentação e nutrição (carga horária maior que 20h) nos últimos 5 anos**	2 (18,2)	0 (0,0)	2 (10,0)
**Participaram de algum curso sobre alimentação e nutrição (carga horária menor que 20h) nos últimos 5 anos**	2 (18,2)	2 (22,2)	4 (20,0)
**Abordam alimentação e nutrição na rotina de atendimento**	11 (100,0)	9 (100,0)	20 (100,0)

Foi possível identificar seis categorias temáticas guiadas pelos núcleos temáticos “princípios”, “recomendações” e “obstáculos”, abordados no Guia, e uma sobre a “responsabilização pela promoção da alimentação adequada e saudável”, que emergiu das falas e se mostrou relevante para o tema abordado neste estudo. 

A Tabela 2 apresenta citações que ilustram as categorias do núcleo temático “Princípios do Guia”. Os participantes expressaram limitações em seu conhecimento sobre alimentação e nutrição e dificuldades na promoção da alimentação adequada e saudável. Demonstraram falta de confiança em orientar em determinadas áreas e evidenciaram a necessidade de apoio de especialistas e mais estudos para lidar com o tema em suas rotinas. Também foi mencionada a necessidade de mais tempo de atendimento para realizar uma orientação adequada. Um profissional citou a falta de parâmetros a respeito do tamanho das porções de alimentos. A redução dos alimentos aos seus nutrientes individuais esteve presente nos relatos, com recomendações agrupando os alimentos segundo suas fontes de macronutrientes. Os alimentos fontes de carboidratos foram frequentemente associados ao conceito de “não saudável”, sem a análise do nível de processamento desses alimentos. Alguns relatos atribuíram a má alimentação ao desconhecimento dos usuários a respeito do conteúdo nutricional dos alimentos. Houve associação da alimentação saudável a restrições alimentares e adoção de uma abordagem prescritiva, acreditando que as orientações alimentares deveriam ser obedecidas pelos usuários. A falta de adesão às condutas passadas por eles foi atribuída a uma falha do usuário em seguir suas orientações por ser uma questão de escolha individual. 

**Tabela 2:** Trechos de citações dos participantes que ilustram o núcleo temático “Princípios do Guia”. Santa Bárbara d’Oeste, 2022 (n=20)

Núcleo Temático: Princípios do Guia Alimentar^a^	
Categorias temáticas	Falas
**Conhecimento sobre alimentação e saúde**	“*E aí necessita de uma orientação alimentar, de dieta, e que a gente tem uma limitação no que consegue explicar, o que consegue orientar, e aí precisamos ter o suporte dos especialistas. (…) E demanda muito tempo para você orientar, de você ter que estudar também um pouco mais sobre como orientar os pacientes, o que pode comer, o que não pode. (...) E nem sempre a gente sabe a parte nutricional de vitaminas, nutrientes, que a pessoa também precisa ter balanceado. (…) A parte de colesterol, triglicérides, os principais alimentos. Tem alimentos que a gente sabe que é um pouco mais gorduroso, tem outros que a gente nem imagina que pode fazer mal. Então é importante ter esse apoio*” (MD11).
	“(...) *Aí criança também, gestante também… me choca mais é essa questão com o diabético... Acho que um pouco na puericultura também, na introdução alimentar, eu acho que a gente perde um pouco, às vezes até por desconhecimento mesmo, de apresentar de uma forma legal, até por conta de tempo também. Eu acho que por falta de treinamento também, um tema que eu não domino, eu não discuto muito, né, eu não falo muito, acho que por aí*” (MD08).
	“*Eu tenho, assim, um tema específico, eu tenho muita dificuldade com o diabético. Esse eu tenho dificuldade mesmo. (...) Ah, obesidade é um tema mais fácil da gente conduzir, né? Talvez as dislipidemias também, né? Mas assim, de doenças crônicas relacionadas à má alimentação, acho que a gente tem dificuldade*” (MD03).
	“*Alimentação pra criança, eu tenho ainda dificuldade* (...) *Criança, é, eu sinto uma dificuldade. (...) Gestantes também, acho que é um grupo que eu gostaria de melhorar, na minha formação, que na faculdade a gente até estuda, mas não é, é ali, né? Bem por cima*” (ENF02).
	“*Mas eu tenho as minhas dificuldades, né? O caso das porções*, né?” (ENF02).
	“*Claro que eu não tenho tanto conhecimento, então eu falo aquilo que eu... Até onde eu consigo*, né?” (ENF06).
**Reducionismo nutricional**	“*Já pula para o almoço, e aí aquele prato, né, que tem muito carboidrato, aí você já vai encontrando os erros alimentares*” (ENF02).
	“*É difícil você pegar um único que se alimenta corretamente. É muito difícil* (...) *Só que aí você vai procurar o que ele come: ‘O que o senhor comeu de manhã?’. ‘Ah, meu pão’. ‘Mas o pão francês?’. ‘É, pão francês*’” (ENF09).
	“*Aqui, comem mal, não sabem o que comer, não sabem o que é carboidrato, o que é proteína, o que é… Não sabem. Não sabem. (...) Tipo, eles não sabem que o pão aumenta o diabetes*” (MD05).
	“*Eu nunca fico falando carboidrato porque às vezes eles não sabem, mas quando eu falo para eles que não pode comer junto arroz e batata, arroz e mandioca, eles: ‘Ah, Não pode?’ (...) Outra coisa que eles ficam surpresos quando eu falo que não pode comer demais farinha, arroz, feijão e farinha. Mas eu vou comer o quê? (...) Então, e às vezes, é coisa básica assim, igual eu falei, né? Então farinha, farinha de mandioca, né? E eles comem tudo, mas porque eles não sabem que é carboidrato*” (MD07).
	“*Paciente com dislipidemia… faço orientação à atividade física regular, caminhadas, controle alimentar… reduzir a ingesta de açúcares, gorduras, farináceos, aumentar a ingesta hídrica, aumentar a ingesta de fibras, verduras, legumes. (...) Basicamente, reduzir gorduras e farináceos*” (MD04).
	“*A gente fala de uma maneira muito geral: evite massas, evite os doces, evite os alimentos com maior quantidade de gordura*” (MD06).
**Autonomia nas escolhas alimentares**	“*Restrição alimentar não é fácil pra ninguém, é uma mudança de hábito*, né?” (MD10).
	“*Geralmente, toda consulta eu explico o que ele pode e o que ele não pode comer*” (MD07).
	“*A reeducação alimentar, ela só vem pra ajudar, né? Quando o paciente entende e acata o que a gente tá falando*” (ENF08).
	“*Então tem muita dificuldade, eles não seguem, você orienta… Mas têm muita dificuldade* [dos usuários seguirem as orientações feitas]” (ENF02).
	“*Eu percebo assim, que a gente fala da alimentação para eles, eles ouvem. (...) Tem uns que aceitam. Tem uns que não aceitam. (...) Mas é bem difícil a adesão mesmo*” (ENF09).
	“*Que primeira coisa que eles* [os usuários] *falam, né? ‘Nossa, agora eu tenho que parar de comer tudo*, né?’” (ENF07).
	“*Eles preferem se empanturrar de insulina do que cuidar da alimentação em casa, sabe? (...) Infelizmente a concepção alopática é muito cultural, então assim, na cabeça deles eu acho que eles pensam dessa forma assim: Ah, mas é, por exemplo, eu não vou passar essa vontade, eu vou comer isso aqui e depois eu aumento a insulina de 5 a 10 unidades, sei lá*” (ENF03).

^a^Princípios do Guia Alimentar: Alimentação é mais que ingestão de nutrientes; Recomendações sobre alimentação devem estar em sintonia com seu tempo; Alimentação adequada e saudável deriva de sistema alimentar socialmente e ambientalmente sustentável; Diferentes saberes geram o conhecimento para a formulação de guias alimentares; Guias alimentares ampliam a autonomia nas escolhas alimentares (10).

Citações que ilustram as categorias definidas segundo as “Recomendações do Guia” estão apresentados na Tabela 3. Os profissionais relataram que orientavam sobre a importância de redução do consumo de alimentos “industrializados”, uma vez que reconheciam que o consumo era alto e prejudicial à saúde. Apesar do termo “ultraprocessado” ter sido pouco mencionado, os entrevistados pareceram reconhecer estes alimentos. Destacaram-se profissionais que referiram que em suas orientações estimulavam o maior consumo de frutas, arroz e feijão e a substituição de embutidos por ovos. Os entrevistados apontaram o prazer envolvido na alimentação e a importância de comer com atenção, das refeições compartilhadas, do ambiente apropriado e do contexto social da alimentação, tanto para adultos quanto para crianças. A influência do contexto em que ocorre a alimentação e das práticas alimentares familiares foram reconhecidas como fatores para promover uma alimentação saudável.

**Tabela 3:** Trechos de citações dos participantes que ilustram o núcleo temático “Recomendações do Guia”. Santa Bárbara d’Oeste, 2022 (n=20)

Núcleo temático: Recomendações do Guia Alimentar^a^	
Categorias temáticas	Falas
**Consumo de ultraprocessados**	“*O que eu mais oriento, agora eu falo e foco bastante, é evitar alimentos industrializados, que aqui eles comem muita mortadela, embutidos, eles gostam bastante. (...) Eu oriento* *mais no geral… evitar os alimentos industrializados, até bolacha de água e sal que eles acham que é saudável, comer e focar mais nas frutas, arroz e feijão*” (ENF02).
	“*Alimentos industrializados, eles ingerem bastante*…” (MD03).
	“*Então acaba que eles consomem mais industrializados, bastante coisa pronta, e pelo menos o que eu observo quando eu questiono*” (MD09).
	“*Eu sempre pego no pé para não ficar comendo muito embutido. (...) Tudo bem, você não consegue comprar carne? Substitui pelo ovo. Porque a salsicha está cheia de sal, de sódio, de realçador de sabor*. ” (ENF04).
Comensalidade	“*Então, isso a gente sabe que a alimentação é um momento de prazer. Você tem que prestar atenção no que você tá comendo, sentindo o prazer de estar se alimentando. Ali, vendo televisão, você fica totalmente disperso. Você acaba comendo mais, às vezes, e não presta atenção. Não tem aquele prazer que a gente sabe que é se alimentar*” (ENF02).
	“*Se o adulto comer, a criança vai comer, entendeu? Na escola mesmo, você vê. A criança na escola é outra alimentação. Por que come? Vê todo mundo comendo, ele simplesmente come*” (ENF07).
	“*Comer entre as famílias, né? (...) Uma importância da alimentação em família, né? Então isso daí é essencial pra mim*” (MD10).

^a^Recomendações do Guia Alimentar: Faça de alimentos in natura ou minimamente processados a base de sua alimentação; Utilize óleos, gorduras, sal e açúcar em pequenas quantidades ao temperar e cozinhar alimentos e criar preparações culinárias; Limite o uso de alimentos processados, consumindo-os, em pequenas quantidades, como ingredientes de preparações culinárias ou como parte de refeições baseadas em alimentos in natura ou minimamente processados; Evite alimentos ultraprocessados; Prefira sempre alimentos in natura ou minimamente processados e preparações culinárias a alimentos ultraprocessados; Comer com regularidade e com atenção; Comer em ambientes apropriados; Comer em companhia (10).

Os “Obstáculos para a adesão às recomendações do Guia” nortearam a definição das categorias cujas citações estão apresentadas na Tabela 4. O menor tempo e o preparo mais simples ou a ausência de preparo dos alimentos ultraprocessados foram compreendidos como uma maior conveniência para os consumidores, ainda que os relatos relacionassem o preparo das refeições caseiras com alimentos mais saudáveis e mais saborosos. Destacaram-se as narrativas sobre a praticidade oferecida pelos ultraprocessados, que, para os entrevistados, superava o esforço necessário para preparar refeições saudáveis, especialmente diante da sobrecarga com outras atividades domésticas e de cuidado. A falta de tempo foi relatada como um fator significativo que impactava a escolha dos alimentos, o que era uma das principais razões que levavam os usuários a priorizarem refeições rápidas e práticas, frequentemente optando por ultraprocessados e fast-food. O alto custo dos alimentos foi identificado como uma barreira importante para a adoção de uma alimentação saudável pelos usuários. Os entrevistados relataram a propensão das pessoas escolherem alimentos mais baratos e de fácil acesso, embora menos nutritivos, como os ultraprocessados. Os profissionais reconheceram as dificuldades financeiras dos usuários como um desafio significativo para a promoção da alimentação adequada e saudável.

**Tabela 4:** Trechos de citações dos participantes que ilustram o núcleo temático “Obstáculos para a adesão às recomendações do Guia”. Santa Bárbara d’Oeste, 2022 (n=20)

Núcleo temático: Obstáculos para a adesão às recomendações do Guia Alimentar^a^	
Categorias temáticas	Falas
Conveniência	“*Imagina você ter que fazer todo dia, cuidar da casa, e ainda cuidar dos netos, e ainda fazer isso* [preparar as refeições], *realmente não é bem fácil*, né? (ENF07).
	“*Eu acho, eu acho que assim, se alimentar de uma maneira mais saudável, dá um pouco mais de trabalho, né, então você tem que lavar uma salada, você tem que fazer um arrozinho, cozinhar um feijão, e assim, não é todo mundo que quer se dispor, né, apesar de ser o mais gostoso, mas isso é hábito, né, é hábito, se aquela família não tem esse hábito, vai do jeito que der, né, a gente vai, às vezes no mercado você olha no carrinho a pessoa, aquele monte de miojo, então você vê, o que que a pessoa vai, é fácil*, né?” (ENF08).
	“*A gente pega alguns casos, tem crianças obesas, de fazer uso de bastante produtos industrializados.(...) Porque é uma coisa mais fácil pros pais, né? Eles não têm que ir pra cozinha preparar, então, chega no mercado e compra*” (ENF06).
	“*Claro, muitos vão pra alimentação, que é rápida, fast-food, bolacha de recheada*” (ENF02).
	“*Então é mais fácil, eu passo no padaria, tá pronto, eu pego e corro*” (ENF03).
	“*Eles priorizam alimentações fáceis, rápidas... ‘Mas o que você comeu hoje?’ ‘Então, ah, só comi um miojo*’” (ENF08).
	“*Mas você vê que hoje, tem pouco tempo, as mães têm pouco tempo porque trabalha, tem que fazer vinte e nove turnos, né, e aí acaba sendo mais fácil, acaba sendo os ultraprocessados, assim, na grande maioria das vezes*” (MD08).
Custo	“*Eu acho que eles acabam consumindo mais industrializados até por conta do valor, e da praticidade e tal.(...) A gente orienta tudo o que é mais saudável, enfim, mas o paciente às vezes não tem condições financeiras para bancar uma alimentação mais saudável*.” (MD09).
	“*Então às vezes a gente até orienta a questão da alimentação balanceada, dos grupos alimentares, mas muitas vezes o que a gente escuta é que não tem condição financeira de ter salada, fruta, legumes, que acabam ficando muito nos carboidratos e raramente proteína*” (MD06).
	“*Tem gente que tem dificuldade na ingestão de verdura, fala que é caro, eu não acho que seja, mas eles falam que é, então é o produto industrializado* [que substitui]” (MD03).
	“*Alimento processado, como você sabe, tem um custo muito inferior e muitos deles dão prioridade a esses alimentos. Então, é mais uma questão, assim, da praticidade, da facilidade de acesso, que eles mesmos referem que muitas vezes inviabiliza o próprio cuidado alimentar. Então, é aquela questão, né, da espada de dois gumes. Tem a facilidade de aquisição, mas tem também a baixa qualidade. Então, isso daí eu percebo que é um dos mais agravantes em relação a crítica mesmo da qualidade dos alimentos*” (MD02).
	“*Então, fica até difícil falar de uma boa alimentação em um paciente que a renda dele é o que ele recebe do bolsa família. Então, esse é um obstáculo preponderante na minha população*” (MD01).

^a^Obstáculos para a adesão às recomendações do Guia Alimentar: Informação; Oferta; Custo; Habilidades culinárias; Tempo; Publicidade (10).

A Tabela 5 expõe citações que refletem uma visão diversificada sobre a responsabilidade compartilhada pela promoção da alimentação adequada e saudável. Houve consenso sobre a necessidade de envolver a equipe, mas também apontaram desafios como a falta de tempo e de conhecimento, insegurança e necessidade de treinamento sobre esse tema. Houve a visão de que o nutricionista era o profissional adequado para realizar essa tarefa, portanto, os outros profissionais faziam a promoção da alimentação adequada e saudável como era possível, diante de suas limitações. Os entrevistados revelaram priorizar orientações médico-curativas diante das demandas dos usuários, e a abordagem da alimentação e nutrição não era uma prática comum na APS. Um relato apontou o potencial das visitas domiciliares como uma estratégia para a promoção da alimentação adequada e saudável.

**Tabela 5:** Trechos de citações dos participantes que ilustram o núcleo temático “Responsabilização pela promoção da alimentação adequada e saudável”. Santa Bárbara d’Oeste, 2022 (n=20)

Núcleo temático: Responsabilização pela promoção da alimentação adequada e saudável	
Categoria temática	Falas
**Responsabilização pela promoção da alimentação adequada e saudável**	“*Não precisa ser só a nutricionista, porque a nutricionista é uma, né? Uma ou duas. E não tá todo dia na comunidade. Não consegue, né? Se dividir tanto assim. Então, poderia passar essa responsabilidade, essa... Não é responsabilidade, esse compromisso, mas com o preparo*” (ENF06).
	“*A gente sabe que o SUS tem questão de gestão de recursos, então, não adianta a gente sonhar só por ideal, de achar que vai ter uma nutricionista por UBS, né? E mesmo porque, nem se tiver, ela não vai dar conta de fazer orientação de todos os pacientes, uma nutricionista. Então, vai acabar que o médico, o enfermeiro, eles vão ter que ter essa orientação também*” (MD06).
	“*Todos nós como equipe temos que ter esse olhar, né, direcionado à nutrição também. É, isso não é sempre feito, né, porque infelizmente a gente acaba direcionando pra aquela patologia momentânea que o paciente vem com queixa, né, e nem sempre é abordado por essa gama de profissionais, né, no qual o paciente é atendido, a questão nutricional. Então, se a gente pudesse realmente ressaltar essa importância, não só do médico, como dos outros profissionais, nesse sentido, eu acho que seria um ganho, né, pra população… (…) quanto mais profissionais participarem disso, dessa promoção, mais ganho a gente vai ter, né, futuramente na população também*” (MD02).
	“*Olha, o que eu consigo agir de forma mais incisiva em relação aos pacientes, eu consigo, dentro das minhas limitações como médico, afinal eu não sou nutricionista*” (MD01).
	“*Eu acho que a gente pode abordar pouco, tento abordar sempre, mas é, tipo, a gente faz de uma maneira, assim, bem superficial, eu acho. E até, talvez, assim, de falta de treinamento também, enfim, eu acho que, sim, faço, mas acho que poderia melhorar e poderia aumentar a demanda, assim, do tanto que eu faço*” (MD08).
	“*Então, a ação da enfermeira é uma ação mais, infelizmente, mais curativa, né? Que já, a hora que já está com o exame alterado, hora que já está com alguma deficiência, assim, por exemplo, já está realmente com alguma lesão no pé, já está com alguma coisa... Infelizmente, não tenho... eu não vejo muita resolutividade, resultado. (...) E é tão simples, é uma coisa tão óbvia, mas eu não consigo passar isso pro paciente* [a orientação alimentar]. (...) *A gente entende isso, isso é frustrante, é isso que frustra a gente, entendeu? É frustrante. Isso é frustrante. E você sabe a sua responsabilidade, né? Não precisa nem falar, mas é uma coisa que frustra demais*” (ENF07).
	“*Mas é um entrave, viu? Falar de alimentação na atenção primária é complicado. (...) Não é tão simples porque você vê que não é um hábito. Eu te contei isso daí pra você ver que não é um hábito o enfermeiro falar*.” (ENF04).
	“*Tudo começa lá na casa do paciente, como é estratégia, vai fazer visita, vamos avaliar, vamos conversar e eu falo para o paciente, às vezes eu faço visita, vou lá ver o que tem na geladeira, se ele me autorizar, é claro, né? Olha, vamos tentar fazer uma alimentação mais adequada? Então eu tenho esse acesso*” (ENF02).
	“*Eu não tenho tempo de trabalhar essa dieta, né? É muito mais fácil pra mim prescrever o medicamento*” (MD03).

## Discussão

Até onde sabemos, este é o primeiro estudo qualitativo com profissionais da ESF sobre a promoção da alimentação adequada e saudável à luz do Guia Alimentar vigente. O estudo se destaca por captar as perspectivas dos participantes e o conhecimento adquirido por meio de suas experiências com a promoção da alimentação adequada e saudável, permitindo a exploração de informações que surgiram das reflexões dos participantes sobre sua vivência, abrangendo seus conhecimentos e práticas. Mesmo após uma década, os conceitos da segunda edição do Guia não estão plenamente incorporados pelas equipes de ESF do município. Vale ressaltar que o período que antecedeu esta pesquisa foi marcado pela pandemia de COVID-19, quando foram privilegiadas as atividades referentes à pandemia e prejudicada a capacitação em outras áreas do município, como a alimentação e nutrição (19). 

Como evidenciado neste estudo, profissionais podem se sentir despreparados para aconselhar sobre alimentação e nutrição (20) e realizarem orientações alimentares superficialmente (21-22). Essa lacuna de conhecimento contribui para dificuldades e inseguranças na condução das orientações alimentares (21). A realização de processos de formação em promoção da alimentação adequada e saudável, voltados tanto para trabalhadores quanto para gestores, é fundamental para viabilizar o verdadeiro empoderamento e promover uma maior autonomia na tomada de decisão (7). Esses processos devem abordar ferramentas para que indivíduos e comunidades desenvolvam a capacidade de autocuidado e atuem sobre os fatores ambientais que influenciam sua saúde (10). O lócus do estudo – a ESF – propõe um modelo de atenção que considera o contexto físico e social, com a ampliação da compreensão dos profissionais sobre as condições de vida e saúde das comunidades, o processo saúde-doença e a importância de intervenções que transcendem as práticas curativas (17). 

Neste estudo, profissionais referiram o agrupamento dos alimentos conforme sua composição nutricional, dando ênfase a nutrientes específicos, em desacordo com o Guia. Essa perspectiva foi amplamente difundida e utilizada em ações de promoção da alimentação adequada e saudável (23) desde a publicação da primeira edição do Guia (9) e tornou-se muito popular, com recomendações de ingestão calórica e de porções de alimentos, conceitos trazidos pelos participantes deste estudo. A ênfase em nutrientes isolados foi observada, especialmente em relação aos carboidratos que, independentemente do nível de processamento, foram vistos como alimentos a serem evitados. Um dos princípios fundamentais do Guia vigente (“Alimentação é mais que ingestão de nutrientes”) enfatiza uma abordagem abrangente dos conceitos de alimentação e saúde, extrapolando as dimensões fisiológicas e biológicas (10) e dialogando com o referencial da Promoção da Saúde. O Guia incorpora os componentes sociais, econômicos, políticos e culturais e enfatiza a promoção da autonomia nas escolhas e práticas alimentares, o engajamento em ações que fortalecem o autocuidado e o diálogo entre indivíduos, profissionais de saúde e gestores, valorizando o respeito às vontades, às diferenças e às subjetividades (1). Também, os participantes associaram alimentação saudável a dietas restritivas e a adoção de abordagens prescritivas, contrariando o que o Guia preconiza. Ao compreender a alimentação de maneira abrangente e sua conexão com a saúde e o bem-estar, o Guia considera, além dos nutrientes e alimentos, suas combinações, formas de preparo e os aspectos culturais e sociais da alimentação (10).

Já o consumo de ultraprocessados (ou “industrializados”) foi notado pelos profissionais com preocupação. O crescente avanço das técnicas de ciência alimentar em escala global resultou em uma maior produção e consumo dos ultraprocessados, inclusive no Brasil (1,24-26) e a presença desses alimentos na rotina dos usuários foi percebida pelos entrevistados. O consumo excessivo desses produtos é um dos principais fatores que contribuem para a epidemia global de obesidade, diabetes e outras doenças crônicas não transmissíveis (1,26-27). 

A conveniência dos ultraprocessados e fast-foods foi percebida como uma estratégia para poupar tempo e reduzir o esforço necessário para cozinhar. De fato, esses produtos são intencionalmente elaborados para serem práticos, exigindo pouco ou nenhum preparo, com longa vida útil e, muitas vezes, não necessitando de utensílios, o que facilita seu consumo em qualquer ambiente (28-29). Os participantes de nosso estudo perceberam a falta de tempo para planejamento e preparo de refeições e o fácil acesso aos ultraprocessados como desafios, em sintonia com o Guia, que reconhece que a desvalorização do preparo dos alimentos, a sobrecarga das tarefas diárias e a maior oferta de ultraprocessados são obstáculos importantes para a adoção de uma alimentação adequada e saudável (10). O preparo das refeições em casa, embora demande tempo, é fundamental para práticas alimentares saudáveis, e isso deve ser acompanhado da divisão equitativa das tarefas domésticas evitando a sobrecarga das mulheres (30). É essencial que profissionais de saúde integrem essas discussões em suas práticas na APS (10).

Nosso estudo apontou o baixo custo e o fácil acesso aos ultraprocessados e a inacessibilidade financeira aos alimentos “saudáveis” pelos usuários como desafios significativos no contexto alimentar atual. A indústria alimentícia projeta e comercializa esses produtos visando maximizar a lucratividade, o que se reflete em sua acessibilidade econômica (29). Esse custo reduzido incentiva o consumo de ultraprocessados em detrimento de opções mais saudáveis (28). No Brasil, uma alimentação com base em alimentos in natura ou minimamente processados ainda é mais econômica que uma dieta baseada em ultraprocessados (31). Entretanto, a estimativa é que os ultraprocessados possam se tornar ainda mais acessíveis que as opções saudáveis até 2026 (32). Não foi observada entre os participantes a abordagem de estratégias junto aos usuários para economizar na aquisição de alimentos in natura e minimamente processados. A compreensão do papel do ambiente alimentar, considerando tanto o acesso físico quanto o acesso financeiro aos alimentos, é fundamental para lidar com as barreiras que os indivíduos enfrentam para adesão às recomendações do Guia (1).

O ato de comer e suas dimensões foram salientados neste estudo. A comensalidade tem um papel crucial na promoção de hábitos alimentares saudáveis (10) e foi reconhecida pelos profissionais como um fator importante, enfatizando as refeições compartilhadas e as práticas alimentares familiares, que têm um efeito relevante nas escolhas alimentares das crianças, moldando seus padrões alimentares (28). Esta perspectiva, apontada pelos entrevistados, alinha-se às diretrizes do Guia, respaldadas por múltiplas evidências científicas, que destacam, por exemplo, o impacto negativo no consumo alimentar de distrações ambientais e atividades realizadas simultaneamente ao ato de comer, como a utilização de televisão, computador ou celular durante as refeições, reduzindo a percepção dos sinais de saciedade (1). Na perspectiva da Promoção da Saúde, a comensalidade envolve a convivência, o bem-viver, o prazer e a felicidade, que são considerados na construção e na vivência da saúde dos indivíduos em seu dia-a-dia (7,10,33). 

É importante destacar a autopercepção dos participantes enquanto agentes da promoção da alimentação adequada e saudável, que reconheciam a importância da colaboração interprofissional e entendiam que essa ação não deve ser atribuída exclusivamente a um único profissional. A implementação da diretriz de promoção da alimentação adequada e saudável é responsabilidade dos gestores e profissionais do SUS, em parceria com outros atores e setores (10) e a responsabilidade pelos cuidados em alimentação e nutrição deve ser compartilhada entre a equipe de APS (6). Contudo, a falta de conhecimento, segurança e capacitação, apontados neste estudo, são desafios que comprometem a eficácia da promoção da alimentação adequada e saudável na APS. Os serviços da APS são frequentemente marcados pela fragmentação da atenção e insuficiência da responsabilização clínica como resultados da organização do trabalho e dos processos decisórios (17). Isso pode resultar na adoção de abordagens farmacêuticas, vistas como mais rápidas e convenientes (34), como encontrado neste estudo. A integralidade é um pilar na construção do SUS, e uma de suas dimensões se refere à prioridade das ações de promoção e prevenção, reforçando uma postura crítica e a rejeição de um modelo de saúde focado na especialização e na medicalização (17).

Como prováveis limitações podemos citar a condução das entrevistas pela responsável técnica pelo serviço de nutrição no município, o que pode gerar um viés, intencional ou não, nas respostas dos participantes. Para a redução deste viés a entrevistadora garantiu a confidencialidade e anonimato, desenvolveu uma boa relação com os participantes e promoveu um clima de respeito e livre de julgamentos. Já a escolha de restringir os participantes do estudo a médicos e enfermeiros se justifica pela responsabilidade destes pelos encaminhamentos excessivos para atendimentos com nutricionistas na atenção especializada e pela relevância deles no aconselhamento alimentar na APS do município.

Conclui-se que as percepções a respeito dos conhecimentos sobre alimentação e saúde, autonomia nas escolhas alimentares, reducionismo nutricional, conveniência e custo divergem das diretrizes estabelecidas pelo Guia. Já as percepções sobre o consumo de ultraprocessados e comensalidade se alinham aos princípios e recomendações do Guia. Considerando a APS um ambiente propício para a promoção da alimentação adequada e saudável, é necessário que os profissionais de saúde estejam adequadamente prontos para essa função. Para além da necessidade de qualificação desses trabalhadores, a implementação de equipes multiprofissionais pode ser uma estratégia para ampliar a oferta de ações de promoção da alimentação adequada e saudável, além de contribuir com a efetiva implementação do Guia na ESF.

## Data Availability

Os dados da pesquisa estão disponíveis em https://doi.org/10.7910/DVN/MZE9PM.
